# Transfer of a Catabolic Pathway for Chloromethane in *Methylobacterium* Strains Highlights Different Limitations for Growth with Chloromethane or with Dichloromethane

**DOI:** 10.3389/fmicb.2016.01116

**Published:** 2016-07-19

**Authors:** Joshua K. Michener, Stéphane Vuilleumier, Françoise Bringel, Christopher J. Marx

**Affiliations:** ^1^Department of Biological Engineering, Massachusetts Institute of TechnologyCambridge, MA, USA; ^2^Department of Organismic and Evolutionary Biology, Harvard UniversityCambridge, MA, USA; ^3^Biosciences Division, Oak Ridge National LaboratoryOak Ridge, TN, USA; ^4^UMR 7156 UNISTRA –CNRS, Université de StrasbourgStrasbourg, France; ^5^Department of Biological Sciences, University of IdahoMoscow, ID, USA; ^6^Institute for Bioinformatics and Evolutionary Studies, University of IdahoMoscow, ID, USA; ^7^Center for Modeling Complex Interactions, University of IdahoMoscow, ID, USA

**Keywords:** horizontal gene transfer (HGT), bioremediation, chloromethane, *Methylobacterium extorquens*, microbial evolution

## Abstract

Chloromethane (CM) is an ozone-depleting gas, produced predominantly from natural sources, that provides an important carbon source for microbes capable of consuming it. CM catabolism has been difficult to study owing to the challenging genetics of its native microbial hosts. Since the pathways for CM catabolism show evidence of horizontal gene transfer, we reproduced this transfer process in the laboratory to generate new CM-catabolizing strains in tractable hosts. We demonstrate that six putative accessory genes improve CM catabolism, though heterologous expression of only one of the six is strictly necessary for growth on CM. In contrast to growth of *Methylobacterium* strains with the closely related compound dichloromethane (DCM), we find that chloride export does not limit growth on CM and, in general that the ability of a strain to grow on DCM is uncorrelated with its ability to grow on CM. This heterologous expression system allows us to investigate the components required for effective CM catabolism and the factors that limit effective catabolism after horizontal transfer.

## Introduction

Chloromethane (CM) is the most abundant organohalide on earth, accounting for roughly 16% of tropospheric chlorine in 2012, and therefore contributes to chloride-catalyzed ozone depletion (World Meteorological Organization, 2014). Sources of CM are mainly natural, such as biomass burning and tropical plants (Yokouchi et al., 2000; Keppler et al., 2005). An abundant electron-rich compound represents a valuable carbon source for a microbe and, as expected, multiple microbial strains have been isolated based on their ability to grow with CM as the sole source of carbon and energy (Hartmans et al., 1986; Doronina et al., 1996; McAnulla et al., 2001; Woodall et al., 2001; Schäfer et al., 2005; Warner et al., 2005; Nadalig et al., 2011; Nadalig et al., 2014). These microbes are estimated to consume approximately one third of the CM produced each year and therefore represent an ecologically significant sink for CM (Keppler et al., 2005).

**FIGURE 1:**
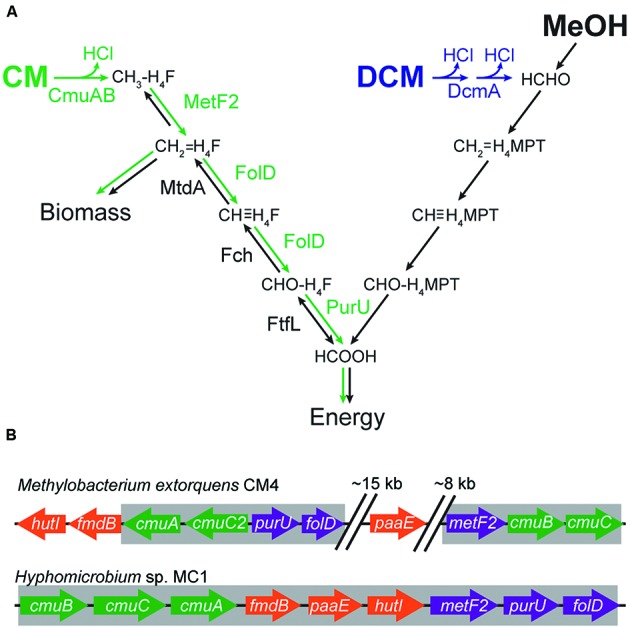
**Genes and enzymes required for growth on C_1_ compounds. (A)** Growth with CM requires a reversal of the flux through the assimilatory tetrahydrofolate (H_4_F) pathway. During growth with methanol (MeOH) or DCM, carbon enters C_1_ metabolism as formaldehyde (HCHO) and is oxidized to formate (HCOOH) in a tetrahydromethanopterin (H_4_MPT)-dependent pathway. Formate is then further oxidized to CO_2_ or reduced in a H_4_F-dependent pathway. During growth with CM, carbon enters as methyl-H_4_F and is oxidized to formate to yield energy. Reversing flux through the assimilatory H_4_F pathway requires three additional enzyme activities (green arrows). **(B)** The *cmu* clusters of *Hyphomicrobium* sp. MC1 is more compact than the corresponding cluster of *M. extorquens* CM4. The regions highlighted in gray were amplified by PCR; cloned into expression plasmids pJM50 and pJM105, respectively; and conjugated into recipient *Methylobacterium* strains.

The model CM-degrading bacterium is *Methylobacterium extorquens* CM4 (hereafter ‘CM4’), an aerobic methylotrophic alpha-proteobacterium (Doronina et al., 1996). Genes necessary for growth on CM have been identified based on insertion mutants of strain CM4 with CM growth defects. In CM4, CM dehalogenation is catalyzed by a pair of proteins, CmuA and CmuB, that transfer the methyl group first to a B_12_ cofactor on CmuA and then to tetrahydrofolate (H_4_F), with concomitant loss of HCl (**Figure 1A**) (Vannelli et al., 1999; Studer et al., 2001). In order to grow with CM as the sole source of carbon and energy, the cell must assimilate a portion of this methyl-H_4_F via methylene-H_4_F and use the rest to generate reducing equivalents. Methylene-H_4_F is also formed during conventional methylotrophic growth, wherein *M. extorquens* oxidizes methanol to formate in a tetrahydromethanopterin (H_4_MPT)-dependent pathway (Chistoserdova et al., 1998; Marx et al., 2003). The formate is then further oxidized to CO_2_ or reduced in a H_4_F-dependent pathway for carbon assimilation (Marx et al., 2003, 2005; Crowther et al., 2008). Since CM methyl groups enter metabolism as reduced methyl-H_4_F, carbon can be assimilated using the same pathways as during growth with methanol. However, the generation of reducing equivalents during growth with CM requires the oxidation of methyl-H_4_F to formate, reversing the flux in this pathway compared to growth with methanol (**Figure 1A**). Three additional enzymes not found in other strains of *M. extorquens*, MetF2, FolD, and PurU, are thought to convert methyl-H_4_F into formate (Studer et al., 2002). However, despite repeated attempts by different researchers in separate laboratories using unique constructs, we have been unable to make targeted mutations in CM4. This limitation has made it difficult to directly test the roles of these accessory genes. The available evidence indicates that *metF2* and *purU* are involved in converting methyl-H_4_F into formate, but the role of *folD* is indeterminate (Vannelli et al., 1998, 1999; Studer et al., 2002).

In addition to CM4, a relatively small and phylogenetically diverse subset of methylotrophs has been found to grow on CM. The genomes of two CM utilizing strains, CM4 and *Hyphomicrobium* sp. MC1 (hereafter ‘MC1’), have been sequenced (Vuilleumier et al., 2011; Marx et al., 2012). In CM4, the CM utilization genes (the *cmu* pathway) are distributed around a large plasmid that also contains genes for cobalamin and folate metabolism. In MC1, the *cmu* genes form a putative operon. The other CM-utilizing strains contain *cmu* pathways with highly homologous enzymes, and their distribution and genetic organization strongly suggest that the pathway has been transferred by horizontal gene transfer (HGT; Nadalig et al., 2014). Horizontal transfer of a complex metabolic pathway can be challenging for the recipient strain, since the transferred pathway must function effectively in its new host, and the host must be able to accommodate the stresses imposed by the new pathway.

We have previously analyzed the factors that limit the effectiveness of a horizontally transferred pathway for catabolism of dichloromethane (DCM), an industrial solvent that differs from CM by only a single chlorine (Michener et al., 2014a). It is unclear how general those factors would be, even for a closely related compound such as CM, since the pathways for catabolism of CM and DCM have different enzymology and metabolic consequences (**Figure 1A**). Catabolism of DCM requires a dedicated cytoplasmic dehalogenase (DcmA) that directly dechlorinates DCM to formaldehyde (La Roche and Leisinger, 1990). As with CM, growth on DCM produces cytoplasmic hydrochloric acid, though twice as much per C_1_ unit. However, DCM is metabolized similarly to the formaldehyde produced during growth on methanol, without requiring the metabolic rerouting necessary for growth on CM (**Figure 1A**). In the case of DCM, expressing DcmA in a variety of other *Methylobacterium* strains initially led to little or no growth (Kayser et al., 2002; Michener et al., 2014b). Effective use of the DCM catabolic pathway required mutations to the host genome that increased chloride efflux (Michener et al., 2014a). Given the similarities and differences between CM and DCM, we wished to understand whether the DCM-utilizing DM4 strain would be preadapted to use CM and, more generally, whether there would be a correlation between the relative ability of a strain to grow with these closely related compounds when provided with the corresponding dehalogenase.

In this work, we have deliberately transferred the CM catabolic pathway into naïve *Methylobacterium* strains, generating new CM-utilizing microbes. We demonstrate that these strains grow poorly on CM, indicating the need for post-transfer refinement. We find no correlation between a strain’s ability to grow with CM and DCM when provided with the corresponding heterologous catabolic pathway. Our heterologous expression system allows facile manipulation, allowing us to measure the fitness effect of accessory genes such as *purU* and *folD*. Finally, we show that growth on CM is not limited by chloride export, in contrast to growth on DCM.

## Results

### Transfer of a *cmu* Cluster from *Hyphomicrobium* sp. MC1, But Not from *M. extorquens* CM4, Enables Diverse *Methylobacterium* Strains to Grow on CM

To reproduce the process of HGT, we cloned the gene clusters implicated in CM catabolism into conjugative plasmids and transferred them into naïve recipient strains (**Figure 1B**). The *cmu* cluster from *Hyphomicrobium* sp. MC1 (Vuilleumier et al., 2011) was cloned as a single insert, yielding pJM105. The *cmu* genes in *M. extorquens* CM4 are dispersed around a large 380 kb plasmid (Marx et al., 2012). Accordingly, we amplified two separate regions of this plasmid, comprising *cmuA*/*folD*/*purU* and *metF2*/*cmuB*/*cmuC*, and combined them into a single insert to construct plasmid pJM50 (**Figure 1B**).

Each of these plasmids was separately introduced into six different recipient strains unable to grow on CM: *M. extorquens* strains AM1 (Peel and Quayle, 1961), PA1 (Knief et al., 2010), DM4 (Gälli and Leisinger, 1985), and BJ001 (Van Aken et al., 2004), as well as *Methylobacterium nodulans* (Sy et al., 2001) and *Methylobacterium radiotolerans* (Sanders and Maxcy, 1979) (Supplementary Table S2). Each of the transconjugants was tested for growth in minimal medium containing CM as the sole source of carbon and energy. After three days of growth, all six of the pJM105 transconjugants containing the *Hyphomicrobium* sp. MC1 *cmu* cluster showed small, but consistent, levels of growth (0.02 < OD_600_ < 0.06), while none of the pJM50 transconjugants reached a comparable optical density. Under these conditions, strain CM4 typically reaches an optical density of ~0.1. Control flasks, containing cells but no CM, did not exceed an OD_600_ of 0.01.

**FIGURE 2:**
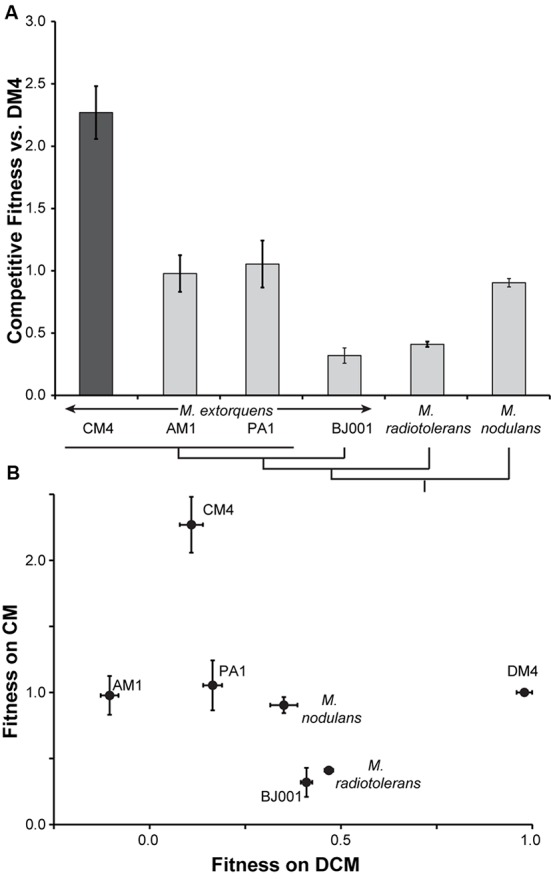
**Fitness during growth on CM and DCM is uncorrelated. (A)** Heterologous expression of *cmu* genes from plasmid pJM105 allows limited growth with CM. Each transconjugant strain, containing pJM105, was individually competed against transconjugant DM4 containing pJM105. As a control, the CM4 strain contained an empty plasmid, pCM62, with the same backbone as pJM105. A simplified phylogenetic tree of the recipient strains is shown below the figure (Michener et al., 2014b). Error bars show one standard deviation, calculated from three biological replicates. **(B)** Fitness during growth on CM and DCM is uncorrelated. CM fitness data are replotted from **(A)**. Data for fitness with DCM are reproduced from Michener et al. (2014b). For growth on DCM, the DCM dehalogenase DcmA was heterologously expressed from a plasmid. This plasmid, pJM10, was conjugated into the same set of recipient strains, and competitive fitness during growth with DCM was measured in a similar fashion as growth with pJM105 and CM. Both axes plot competitive fitness of a given recipient relative to the corresponding DM4 transconjugant.

Poor growth of the pJM105 transconjugants made it difficult to accurately quantify growth rates and yields, so instead we characterized their growth based on competitive fitness. Each of the transconjugants, as well as *M. extorquens* CM4 as a positive control, was mixed with the transconjugant of DM4 and grown with CM as the sole source of carbon and energy. We measured the population sizes and population ratios before and after growth, and then calculated the competitive fitness relative to DM4 (**Figure 2A**). As expected, the fitness of the native CM-consuming strain CM4 was significantly higher than any of the transconjugants (*p* < 0.01 for all transconjugants, two-tailed *t*-test). However, each of the transconjugants had non-zero fitness, indicating that they grew with CM as the sole source of carbon and energy. For comparison, we also competed AM1, PA1, and DM4 against CM4 directly. These competitions confirmed that the transconjugant strains grow with CM, but at 23-44% of the fitness of CM4 (Supplementary Figure S1).

### Effectiveness of CM Catabolism Does Not Correlate with DCM Use across *Methylobacterium* Strains

We previously measured the fitness of this same set of recipient strains during growth on DCM after introduction of a heterologous DCM catabolic pathway (Michener et al., 2014b). Comparing the fitness of the strains on CM and DCM, we find no correlation between an individual’s fitness on CM and DCM (linear regression, *p* = 0.49, **Figure 2B**).

### Deletions in the *cmu* Gene Cassette Allow Identification of Genes Essential for Growth with CM in *M. extorquens* AM1

**FIGURE 3:**
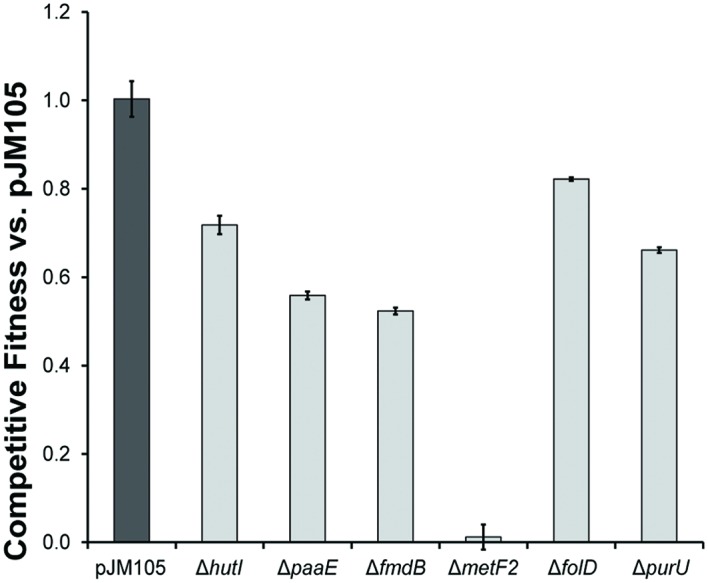
**Gene deletions identify fitness effects of accessory genes.** Each accessory gene in the gene cassette of pJM105 was individually deleted by inverse PCR with primer pairs designed to overlap and create a clean deletion (Supplementary Table S1). The fitness cost of each deletion was determined by competing a strain containing the mutant plasmid against an otherwise isogenic strain containing pJM105.

Each of the six accessory genes in the *cmu* gene cassette, *metF2*, *purU*, *folD*, *paaE*, *hutI*, and *fmdB* was individually deleted from pJM105, and the modified plasmids were introduced into AM1. We determined the fitness effect of each single-gene deletion by competing strains containing the modified plasmids against a strain containing the original plasmid during growth with CM (**Figure 3**). Only one gene, *metF2* encoding a methylene H_4_F reductase (**Figure 1**), was essential for growth on CM, while the other deletions imposed fitness costs of 18–47%.

### Chloride Transport Does Not Limit Growth with CM

**FIGURE 4:**
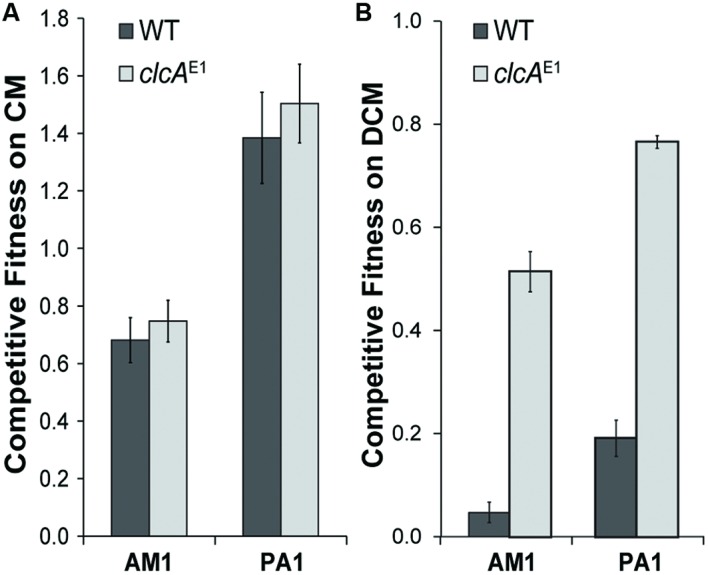
**ClcA overexpression improves fitness during growth with DCM but not with CM. (A)** Growth with CM is unaffected in ClcA overexpression. Plasmid pJM105 was conjugated into two *M. extorquens* strains, AM1 and PA1, both in the wild type strain and a mutant that overexpresses ClcA. These transconjugants were then competed against DM4 + pJM105. The fitness of the wild type and mutant are indistinguishable in both cases. Error bars show one standard deviation, calculated from three biological replicates. **(B)** In contrast, ClcA overexpression significantly increases fitness during growth with DCM of transconjugants containing pJM10. Data in part B are reproduced from Michener et al. (2014a).

We previously showed that growth of transconjugant *Methylobacterium* strains with DCM was limited by the need to export the chloride ions produced as a byproduct of dechlorination (Michener et al., 2014a). Mutations that increased chloride efflux, such as overexpression of the ClcA chloride:proton antiporter, significantly increased fitness during growth on DCM (**Figure 4B**). Accordingly, we tested whether ClcA overexpression would increase the fitness of a *Methylobacterium* strain during growth with CM. We introduced the pJM105 plasmid into mutant strains of AM1 and PA1 that each overexpress ClcA. In both cases, the fitness of the ClcA overexpression strain was indistinguishable from an otherwise isogenic control (**Figure 4**).

## Discussion

### Heterologous CM Use Does Not Correlate with DCM Use across *Methylobacterium* Strains

As with growth on DCM, the ability to exploit this horizontally transferred pathway is common and all of the recipients were able to grow on CM. Consistent with our previous results, the phylogenetic relationships between recipients was not predictive of their fitness, though we might expect less of a phylogenetic effect since the heterologous pathway was transferred from outside the genus. Additionally, transfer of the pathway allowed only limited growth, ranging from 14 to 46% of the fitness of a natural isolate (**Figure 2A**). Despite these general similarities, the lack of correlation between fitness on CM and DCM suggests that the fitness-limiting factors are different for the catabolic pathways of these two chlorinated methanes. We assume in our interpretation of the competition experiments that the strains compete solely through consumption of the carbon source. Any other competitive interactions would likely have similar effects during growth both with CM and with DCM.

### Gene Deletions Demonstrate That *metF2*, But Not *purU* or *folD*, Is Essential for Growth with CM in *M. extorquens* AM1

The MetF2 enzyme was essential for growth of AM1 with CM, as had previously been shown for CM4 (Studer et al., 2002). CM4 has two 5,10-methylene-H_4_F reductase gene homologs: a chromosomal *metF* shared by strain AM1 (99.7% amino acid identity) and a plasmid-borne *metF2* that is part of the *cmu* cluster and shares only 26% amino acid identity with the chromosomal homolog. *Hyphomicrobium* sp. MC1 also contains two *metF* homologs, *metF* with 65.2% amino acid identity to the CM4 *metF* and *metF2* with 28.4% amino acid identity to the CM4 *metF2*. Since the native AM1 *metF* is unable to sustain growth with CM in the absence of *metF2* from *Hyphomicrobium* sp. MC1, we conclude that the chromosomal *metF* is either misregulated during growth on CM or is incapable of catalyzing the oxidative reaction with the necessary flux.

The partial fitness defect of the Δ*purU cmu* gene cassette contrasts with previous findings showing that this gene was essential for growth of CM4 with CM (Vannelli et al., 1999). We hypothesize that the formate-H_4_F ligase FtfL is capable of catalyzing a limited flux from formyl-H_4_F to formate. In a host such as AM1 with low CM flux, the reduction in flux from a *purU* deletion would likely only produce a small but measurable fitness cost. Reduction to a similar absolute level of flux in CM4 might either reduce growth to a level that gives the appearance of essentiality or lead to increased accumulation of one or more toxic intermediates.

The observed fitness cost of the *folD* deletion in the *cmu* cassette provides the first evidence that this enzyme plays an important role in CM catabolism. As with *purU*, however, the fact that the *folD* knockout still grows on CM strongly suggests that MtdA and Fch can carry C_1_ flux in the oxidative direction (**Figure 1A**), albeit at levels insufficient for the Δ*folD* strain to match even the limited growth of AM1 with the intact *cmu* gene cluster. Indeed, previous work replacing MtdA and Fch with FolD in AM1 has shown that FolD is sufficient for growth on succinate, which requires relatively little flux through the H_4_F pathway, but is insufficient for growth on methanol, which requires a much higher flux (Marx and Lidstrom, 2004). Both FolD and MtdA/Fch appear to poorly catalyze the reverse reaction, presumably due either to enzyme biochemistry or to allosteric regulation (Martinez-Gomez et al., 2013). More broadly, these results are consistent with past work showing that the phenotype of lesions in C_1_ metabolic pathways can vary dramatically between different environments depending on the level of flux through the pathway (Marx et al., 2003; Nayak and Marx, 2014).

We have not investigated the fitness effects of deleting *cmuA*, *cmuB*, or *cmuC*, each of which was previously reported to be required for growth of CM4 with CM (Vannelli et al., 1999).

### Accessory Genes *hutI*, *paaE*, and *fmdB* Are Beneficial during Growth with CM in *M. extorquens* AM1

Based on operon structure and gene conservation, *paaE*, *hutI*, and *fmdB* were predicted to be involved in growth with CM. However, this work is the first direct demonstration that these genes are beneficial during growth with CM. None of the genes were necessary for growth with CM, but deleting them imposed fitness costs of 25-47%. Further work will be needed to elucidate the precise contribution from each of these accessory genes.

These deletion experiments may also explain our inability to productively transfer the *cmu* operon from its best-studied host, *M. extorquens* CM4, into closely related *Methylobacterium* strains. The gene clusters cloned from strains MC1 and CM4 differ in four genes (**Figure 1B**): gene *cmuC2* is only found in the CM4 cluster, while genes *fmdB*, *paaE*, and *hutI* were not included in the CM4 cluster that we cloned. Two of these genes, *fmdB* and *hutI* are immediately adjacent to *cmuA* in *M. extorquens* CM4 and the third gene, *paaE*, occurs on the native CM4 plasmid pCMU01 roughly equidistant between the two segments that we amplified (**Figure 1B**). A simple multiplicative model based on our deletion studies, which assumes each mutation has a constant proportional effect on fitness regardless of the genetic background, would predict a fitness of ~20% for the triple deletion Δ*fmdB*Δ*hutI*Δ*paaE* relative to the full pJM105 plasmid. If introduction of plasmid pJM50, which lacks *fmdB*, *hutI*, and *paaE*, allowed strains to grow with CM, but only 20% as effectively as with pJM105, we would not have been able to detect the growth of pJM50 transconjugants. Additionally, the *cmu* cluster that we cloned from *Hyphomicrobium* sp. MC1 has the native gene order and spacing, unlike the CM4 cluster, and this may favor heterologous *cmu* gene expression. In our single-gene deletion experiments, the start and stop codons of the deleted gene were preserved to minimize polar effects on the remainder of the gene cluster. However, even such conservative deletions may affect transcript stability, and we cannot rule out fitness effects due to disruption of undetected genes in the gene cluster or truncated polypeptides resulting from the deletions.

While it is possible that complete transfer of the native 380 kb CM4 plasmid might suffice for effective growth with CM, attempts to transfer the entire plasmid into naïve recipients has not been successful. We also note that several other genes are present on the native CM4 plasmid, are specifically induced during growth on CM, were not transferred in our experiment, and therefore are not essential for CM catabolism in *Methylobacterium* strains (Roselli et al., 2013). We have shown in this work that *hutI*, *paaE*, and *fmdB* are beneficial, but not essential, during growth with CM. The additional CM-induced genes from the native CM4 plasmid may provide a similar fitness benefit during growth on CM. Our heterologous expression system offers a unique opportunity to explore these questions in a genetically tractable system such as *M. extorquens* AM1.

### Chloride Transport Does Not Limit Growth with CM in AM1 or PA1

Growth with CM and DCM both require dechlorination, yet growth with DCM is dependent on the level of chloride efflux while growth with CM appears unaffected, based on our ClcA overexpression experiments. Growth on DCM was highly sensitive to the *clcA* expression level, with a twofold change in *clcA* providing a roughly four-fold change in fitness (Michener et al., 2014a). The chloride burden of growth on CM is half that of growth on DCM, so the native chloride export capacity of the recipients may suffice for CM growth. In combination with our observation that the fitness of a given recipient strain during growth on DCM is not predictive of its fitness during growth on CM, we conclude that growth with DCM and CM place different stresses on the cell. However, we note that both the *cmu* operon in *Hyphomicrobium* sp. MC1 and the pCMU01 plasmid in CM4 also contain a second copy of *clcA* that we chose not to include in our heterologous operons. The factors limiting growth on CM in *Methylobacterium* strains may differ from those in *Hyphomicrobium* strains, with native chloride export capacity potentially higher in the *Methylobacterium* strains.

Having demonstrated that chloride export mediated by ClcA does not limit heterologous CM growth, we propose several alternate hypotheses for the limited transconjugant fitness during growth on CM. First, growth on CM is strongly dependent on cobalamin and folate, and the large CM4 plasmid contains 16 genes involved in B_12_ metabolism in addition to *Methylobacterium* core *cob* genes, as well as seven genes involved in folate metabolism, 6 of which do not have chromosomal homologs (Roselli et al., 2013). If chromosomally encoded cobalamin and folate metabolism is inefficient at providing the cofactors essential for dehalogenation, it would limit CM growth in the other *Methylobacterium* strains. Second, growth with CM introduces carbon at an unusual branch point in the *Methylobacterium* C_1_ metabolic network (Nayak and Marx, 2014), requiring the reversal of a metabolic pathway that more commonly functions in the reductive direction during growth with methanol or DCM (**Figure 1A**). Therefore, the recipients may be misregulating their metabolic networks for the new demands placed upon them during growth with CM. Third, the transfer of a nine-gene pathway between microbial families may affect protein expression levels in the new genetic environment, with significant costs to pathway flux and organismal fitness (Chou et al., 2014). Experimental evolution of these transconjugants, selecting for increased fitness during growth on CM, will help to investigate these hypotheses (Michener et al., 2014a; Clark et al., 2015).

## Materials and Methods

### Media and Chemicals

All chemicals were purchased from Sigma-Aldrich (St. Louis, MO, USA) unless otherwise noted. *Escherichia coli* were grown at 37°C in LB broth. *Methylobacterium* strains were grown in M-PIPES at 30°C, supplemented with 3.5 mM succinate for routine growth and with 12.5 μg/mL tetracycline as needed (Delaney et al., 2013). For CM growth, *Methylbacterium* strains were diluted to an OD_600_ of 0.001 in 10 mL of M-PIPES without tetracycline in 50 mL glass flasks sealed with silicone rubber stoppers. A gas-tight syringe was then used to transfer 1 mL of CM from a gas sampling bulb maintained at 16 psig into the headspace of the sealed flask. Cultures were grown aerobically for three days before analysis.

### Plasmid Construction and Matings

Genomic DNA from *Hyphomicrobium* sp. MC1 and *M. extorquens* CM4 were prepared using the Wizard Genomic DNA Purification Kit (Promega, Madison, WI, USA). To construct plasmid pJM50, two genomic regions were amplified from *M. extorquens* CM4 gDNA using the Q5 polymerase with the high GC enhancer (New England Biolabs, Ipswich, MA, USA) (Supplementary Table S3). A single 6.2 kb amplicon contained *cmuA*, *purU*, and *folD*, while a second 3.6 kb amplicon contained *metF*, *cmuB*, and *cmuC*. The amplicons were combined in the pCM62 plasmid backbone using Gibson assembly (NEB) and transformed into chemically competent 10β *E. coli* (NEB). To construct plasmid pJM105, a single genomic region was amplified from *Hyphomicrobium* sp. MC1 gDNA as three overlapping amplicons of 2.6, 4.2, and 3.6 kb and assembled as for pJM50. Plasmids were mated from the 10β cloning *E. coli* strain into recipient *Methylobacterium* strains by tri-parental matings as described previously (Fulton et al., 1984).

To construct the deletion plasmids, pJM107-112, plasmid pJM105 was amplified by inverse PCR with primers designed to overlap and create a clean deletion. The desired open reading frame was truncated, deleting the majority of the gene while leaving the start and stop codons intact and in-frame to minimize polar effects. The resulting amplicons were circularized using Gibson assembly, transformed into chemically competent *E. coli*, and mated into *M. extorquens* AM1 as described above.

### Competitive Fitness Assays

Fitness assays were performed largely as described previously (Lee et al., 2009). In brief, cultures were grown in M-PIPES containing succinate and tetracycline, then diluted to OD 0.01 in fresh M-PIPES containing CM. After two days of growth, the cultures were mixed with the appropriate competitor strain, diluted into fresh M-PIPES containing CM, and grown for a further three days. Pre-growth population samples were frozen at -80°C for later analysis. After competitive growth in mixed culture, the population ratios in the mixed culture both before and after growth were determined by flow cytometry (Michener et al., 2014a). Population sizes were determined based on optical density at 600 nm.

To measure the fitness of the pJM50 and pJM105 transconjugants, strains were competed against *M. extorquens* DM4 *ΔdcmA* Venus containing the appropriate plasmid (Michener et al., 2014a) or against *M. extorquens* CM4 containing an empty tetracycline plasmid, pCM62 (Marx and Lidstrom, 2001). To measure the fitness of the deletion plasmid transconjugants, the deletion plasmids were conjugated into *M. extorquens* AM1 *Δcel* Venus and competed against *M. extorquens* AM1 *Δcel* Cherry + pJM105. To measure the fitness of the chloride transport mutants, pJM105 was conjugated into *M. extorquens* PA1 *Δcel* mCherry *clcA*^E1^ and *M. extorquens* AM1 *Δcel* mCherry *clcA*^E1^ and competed against *M. extorquens* DM4 *ΔdcmA* Venus + pJM105 (Michener et al., 2014b).

## Author Contributions

All authors contributed to the design of experiments, the interpretation of data, and drafting of the manuscript. Experiments were performed by JM.

## Conflict of Interest Statement

The authors declare that the research was conducted in the absence of any commercial or financial relationships that could be construed as a potential conflict of interest.
