# Supplementary material for: Transfer of a Catabolic Pathway for Chloromethane in Methylobacterium Strains Highlights Different Limitations for Growth with Chloromethane or with Dichloromethane
Source: Front Microbiol. 2016 Jul 19;7:1116. doi: 10.3389/fmicb.2016.01116 (PMC4949252; doi:10.3389/fmicb.2016.01116)
Supplement: Supplementary file 1 [file Table_1.DOCX]

Supplementary Material

**Effectiveness of Heterologous Catabolism of Chloromethane and Dichloromethane Are Uncorrelated in *Methylobacterium* Strains**

**Joshua K. Michener*, Stéphane Vuilleumier, Françoise Bringel, and Christopher J. Marx**

*** Correspondence:** Corresponding Author: michenerjk@ornl.gov

Supplementary Table 1: Plasmids used in this study

| **Plasmid** | **Description** | **Genomic DNA source** | **Reference** |
| --- | --- | --- | --- |
| pCM62 | *colE1 oriVT(IncP) traJ’ trfA tetAR* |  | Marx and Lidstrom, 2001 |
| pJM50 | pCM62 *cmuAC2 purU folD metF cmuBC* | *M. extorquens* CM4 | This work,  GenBank KT950470 |
| pJM105 | pCM62 *cmuBCA fmdB paaE hutI metF purU folD* | *Hyphomicrobium* sp. MC1 | This work,  GenBank KT950471 |
| pJM107 | pJM105 Δ*hutI* | *Hyphomicrobium* sp. MC1 | This work |
| pJM108 | pJM105 Δ*paaE* | *Hyphomicrobium* sp. MC1 | This work |
| pJM109 | pJM105 Δ*fmdB* | *Hyphomicrobium* sp. MC1 | This work |
| pJM110 | pJM105 Δ*metF2* | *Hyphomicrobium* sp. MC1 | This work |
| pJM111 | pJM105 Δ*folD* | *Hyphomicrobium* sp. MC1 | This work |
| pJM112 | pJM105 Δ*purU* | *Hyphomicrobium* sp. MC1 | This work |
