# Supplementary material for: Transfer of a Catabolic Pathway for Chloromethane in Methylobacterium Strains Highlights Different Limitations for Growth with Chloromethane or with Dichloromethane
Source: Front Microbiol. 2016 Jul 19;7:1116. doi: 10.3389/fmicb.2016.01116 (PMC4949252; doi:10.3389/fmicb.2016.01116)
Supplement: Supplementary file 2 [file Table_2.DOCX]

Supplementary Material

**Effectiveness of Heterologous Catabolism of Chloromethane and Dichloromethane Are Uncorrelated in *Methylobacterium* Strains**

**Joshua K. Michener*, Stéphane Vuilleumier, Françoise Bringel, and Christopher J. Marx**

*** Correspondence:** Corresponding Author: michenerjk@ornl.gov

Supplementary Table 2: Strains used in this study

| **Designation** | **Strain** | **Genotype** | **Reference** |
| --- | --- | --- | --- |
| AM1 | CM3120 | *M. extorquens* AM1 Δ*cel* *katA*::*mCherry* | Chubiz *et al.*, unpublished |
| PA1 | CM3839 | *M. extorquens* PA1 Δ*cel* *hpt*::*mCherry* | Nayak *et al.*, unpublished |
| DM4 | CM4250 | *M. extorquens* DM4 Δ*dcmA* *hpt*::*Venus* | Michener et al., 2014a |
| CM4 | CM4 | *M. extorquens* CM4 | Doronina et al., 1996 |
| BJ001 | BJ001 | *M. extorquens* BJ001 | Van Aken et al., 2006 |
| *M. nodulans* | ORS 2060 | *M. nodulans* ORS 2060 | Sy et al., 2004 |
| *M. radiotolerans* | JCM 2831 | *M. radiotolerans* JCM 2831 | Sanders et al., 1979 |
| AM1-C *clcA*^E1^ | CM4383 | *M. extorquens* AM1 Δ*cel* *katA*::*mCherry clcA*Δ*(-66→-55)* | Michener et al., 2014b |
| PA1-C *clcA*^E1^ | CM4385 | *M. extorquens* PA1 Δ*cel* *hpt*::*mCherry clcA*Δ*(-66→-55)* | Michener et al., 2014b |
