# Supplementary material for: Transfer of a Catabolic Pathway for Chloromethane in Methylobacterium Strains Highlights Different Limitations for Growth with Chloromethane or with Dichloromethane
Source: Front Microbiol. 2016 Jul 19;7:1116. doi: 10.3389/fmicb.2016.01116 (PMC4949252; doi:10.3389/fmicb.2016.01116)
Supplement: Supplementary file 3 [file Table_3.DOCX]

Supplementary Material

**Effectiveness of Heterologous Catabolism of Chloromethane and Dichloromethane Are Uncorrelated in *Methylobacterium* Strains**

**Joshua K. Michener*, Stéphane Vuilleumier, Françoise Bringel, and Christopher J. Marx**

*** Correspondence:** Corresponding Author: michenerjk@ornl.gov

Supplementary Table 3: Primers used in this study

| **Primer** | **Sequence (5’ to 3’)** | **Use** |
| --- | --- | --- |
| cmuA FWD | TGTGAGCGGATAACAATTTCACACAGGAAACAGCTAGACGATAGGTTCAGCG | Cloning of pJM50 |
| cmuA REV | CCAGGGATTGAGAGATCGGCATGCGATGGAGAGAATAG | Cloning of pJM50 |
| metF FWD | CTATTCTCTCCATCGCATGCCGATCTCTCAATCCCTGG | Cloning of pJM50 |
| metF REV | TCAGCGGGTGTTGGCGGGTGTCGGGGCTGGCTTAACTCATCGCCTCAGAGC | Cloning of pJM50 |
| folD FWD | TGTGAGCGGATAACAATTTCACACAGGAAACAGCTCTCAAAGACCAGGCATTTCACTTAC | Cloning of pJM105 |
| MC1 midR2 | GCTTTCGCTGGTTCCCGAAATC | Cloning of pJM105 |
| MC1 midF2 | GAAGGCGAATTGCGTCACGATTTC | Cloning of pJM105 |
| MC1 midR3 | TTTGAAGGCGATGATTGAGACATGC | Cloning of pJM105 |
| MC1 midF3 | GATATTTGATGGACGCGCATGTC | Cloning of pJM105 |
| cmuB REV | TTCAAGGGAAATGTCGACGCAGAAC | Cloning of pJM105 |
| hutI FWD | CGCTTCCAGAAGAAAATGTTGCGTCGATTATGAC | Cloning of pJM107 |
| hutI REV | ACATTTTCTACTGGAAGCGATCCAAGGAATAAC | Cloning of pJM107 |
| fmdB FWD | TCAAATGGATATCGCAGCGGAACTGATAAAG | Cloning of pJM109 |
| fmdB REV | CCGCTGCGATATCCATTTGACCAAGCCAAATCCC | Cloning of pJM109 |
| paaE FWD | TCCGACATTTCGTTCAACACGCAAGGAGTAA | Cloning of pJM108 |
| paaE REV | GTGTTGAACGAAATGTCGGAGGCGAAGAAAC | Cloning of pJM108 |
| metF FWD | ATCCCTTTAAAGGAAGAAGTTCGATTGAGATATTGC | Cloning of pJM110 |
| metF REV | ACTTCTTCCTTTAAAGGGATAGATCTGCATTTCTTCC | Cloning of pJM110 |
| folD FWD | TGACGAGAAG ATAACGCTTCTCCAGAGATTCAAC | Cloning of pJM111 |
| folD REV | GAAGCGTTAT CTTCTCGTCAACACAGTCAATTC | Cloning of pJM111 |
| purU FWD | ATTCGTTCAATTAGATCTTGGCTGGAGAAGAAGTTTG | Cloning of pJM112 |
| purU REV | CAAGATCTAATTGAACGAATTTCGCATCTGGATTC | Cloning of pJM112 |
