# Supplementary material for: Transfer of a Catabolic Pathway for Chloromethane in Methylobacterium Strains Highlights Different Limitations for Growth with Chloromethane or with Dichloromethane
Source: Front Microbiol. 2016 Jul 19;7:1116. doi: 10.3389/fmicb.2016.01116 (PMC4949252; doi:10.3389/fmicb.2016.01116)
Supplement: Supplementary file 4 [file Image_1.PDF]

Supplementary Material

Effectiveness of Heterologous Catabolism of Chloromethane and Dichloromethane Are Uncorrelated in *Methylobacterium* Strains

Joshua K. Michener*, Stéphane Vuilleumier, Françoise Bringel, and Christopher J. Marx

*** Correspondence:** Corresponding Author: michenerjk@ornl.gov

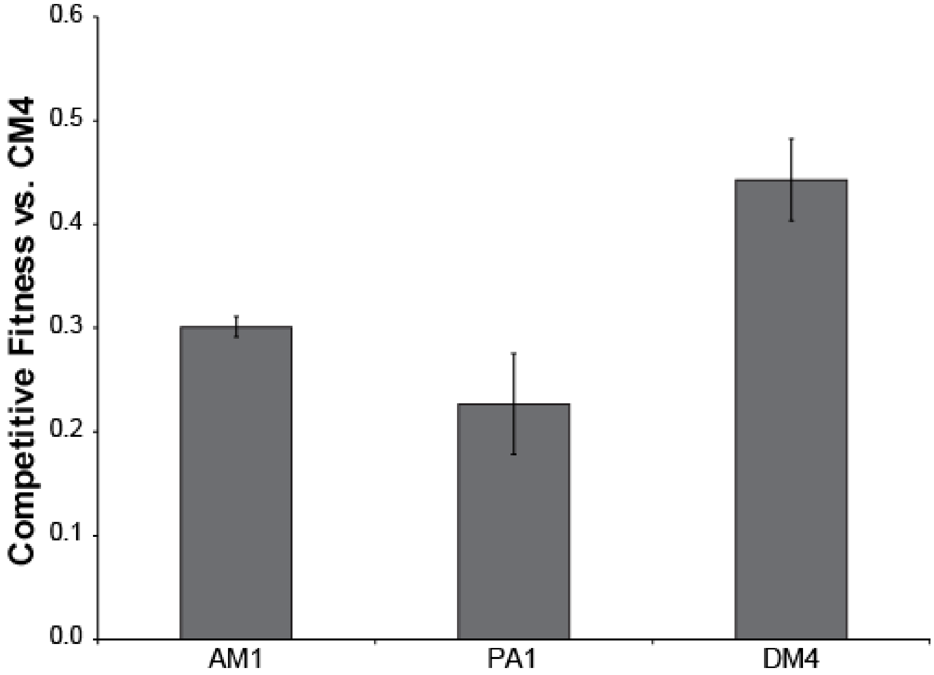

Supplementary Figure 1: Transconjugants are less fit than CM4 during competitive growth with CM. The three fluorescently-labeled *M. extorquens* transconjugants, each containing pJM105, were separately competed against *M. extorquens* CM4 containing an empty plasmid. Each pJM105 transconjugant grew with CM, though with only 23-44% of the fitness of the native CM-degrading strain.
